# Factors influencing mass drug administration adherence and community drug distributor opportunity costs in Liberia: a mixed-methods approach

**DOI:** 10.1186/s13071-021-05058-w

**Published:** 2021-10-28

**Authors:** Efundem Agboraw, Fred Sosu, Laura Dean, Alice Siakeh, Rachael Thomson, Karsor Kollie, Eve Worrall

**Affiliations:** 1 Department of Vector Biology, Liverpool School of Tropical Medicine, Pembroke Street, Liverpool, L3 5 QA UK; 2 University of Liberia, Pacific Institute for Research and Evaluation (UL-PIRE), Capitol Hill, Monrovia, Liberia; 3 Department of International Public Health, Liverpool School of Tropical Medicine, Pembroke Street, Liverpool, L3 5 QA UK; 4 Department of Tropical Disease Biology, Liverpool School of Tropical Medicine, Pembroke Street, Liverpool, L3 5 QA UK; 5 Ministry of Health, SKD Boulevard, Monrovia, Liberia

**Keywords:** Neglected diseases, MDA, Adherence, Opportunity cost, CDD, Community, Liberia

## Abstract

**Background:**

Preventive chemotherapy delivered via mass drug administration (MDA) is essential for the control of neglected tropical diseases (NTDs), including lymphatic filariasis (LF), schistosomiasis and onchocerciasis. Successful MDA relies heavily on community drug distributor (CDD) volunteers as the interface between households and the health system. This study sought to document and analyse demand-side (households) and supply-side (health system) factors that affect MDA delivery in Liberia.

**Methods:**

Working in two purposively selected counties, we conducted a household MDA access and adherence survey; a CDD survey to obtain information on direct and opportunity costs associated with MDA work; an observational survey of CDDs; and key informant surveys (KIS) with community-level health workers. Data from the CDD survey and Liberian minimum wage rates were used to calculate the opportunity cost of CDD participation per MDA round. The observational data were used to calculate the time spent on individual household-level tasks and CDD time costs per house visited. KIS data on the organisation and management of the MDA in the communities, and researcher reflections of open-ended survey responses were thematically analysed to identify key demand- and supply-side challenges.

**Results:**

More respondents were aware of MDA than NTD in both counties. In Bong, 39% (103/261) of respondents reported taking the MDA tablet in the last round, with “not being informed” as the most important reason for non-adherence. In Maryland, 56% (147/263) reported taking MDA with “being absent” at the time of distribution being important for non-adherence. The mean cost per CDD of participating in the MDA round was −$11.90 (median $5.04, range −$169.62 to $30.00), and the mean time per household visited was 17.14 min which equates to a mean opportunity cost of $0.03 to $0.05 per household visited. Thematic analysis identified challenges, including shortages of and delays in medicine availability; CDD frustration over costs; reporting challenges; and household concerns about drug side effects.

**Conclusions:**

Improved adherence to MDA and subsequent elimination of NTDs in Liberia would be supported by an improved medicine supply chain, financial compensation for CDDs, improved training, healthcare workforce strengthening, greater community involvement, capacity building, and community awareness.

**Graphical Abstract:**

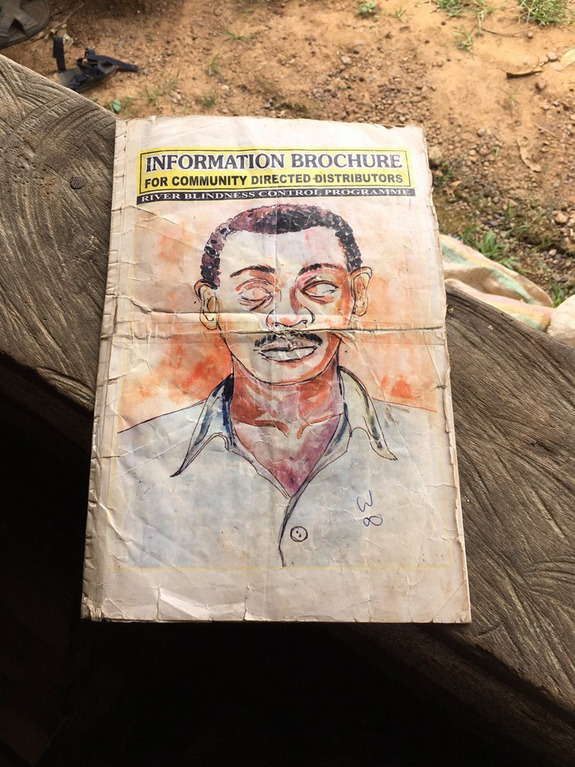

**Supplementary Information:**

The online version contains supplementary material available at 10.1186/s13071-021-05058-w.

## Background

Neglected tropical diseases (NTDs) are a group of 20 diseases that have historically received scant attention and are concentrated among individuals with very low incomes [1, 2]. Preventive chemotherapy delivered via mass drug administration (MDA) is essential for the control of NTDs including lymphatic filariasis (LF), schistosomiasis and onchocerciasis [2]. Low and/or inequitable MDA coverage challenges NTD program effectiveness, the achievement of the new 2030 NTD elimination targets [3–5], and the overarching goal of universal health coverage [6]. Heavy reliance on drug donations and volunteers such as community drug distributors (CDDs) threatens MDA sustainability [1, 3].

Liberia is situated on the west coast of Africa bordered by Sierra Leone, Guinea, Cote d'Ivoire and the Atlantic Ocean, with a population of 5.05 million people. Onchocerciasis, LF and schistosomiasis are endemic throughout Liberia and are the most common infections among its poorest communities [7, 8]. Guided by their NTD Master Plan, the Liberian Ministry of Health and partners sought to eliminate LF, onchocerciasis and schistosomiasis by 2020 [9, 10] through a combination of annual MDA with ivermectin (IVM) and albendazole (ABD); disability management and inclusion; and vector control [7].

The national NTD program led its first 3 yearly MDA campaigns with IVM and ABD in 2012–2013 achieving 82% coverage [11]. Household acceptability and adherence to MDA and the volunteering efforts of CDDs made this possible. CDDs have been at the forefront of drug (and bed net) distribution since 2012, with some individual CDDs having over 10 years of experience. The Ebola virus disease (EVD) outbreak in Liberia (2014–2015) severely disrupted public health programs for NTDs including MDA [12]. Following containment of the EVD outbreak in 2015, the Liberian Ministry of Health and partners restarted MDA in 2017 [12].

CDDs are considered the primary care providers at the community level. However, health system and program-related factors such as inadequate training and supervision of CDDs by health facility staff have an adverse impact on CDD performance [9, 10]. CDDs also incur direct (financial expenditure) and opportunity costs (indirect costs) for their participation in MDA programs. Opportunity cost is defined as the cost of the next-best opportunity foregone, and in the case of CDDs, this is time that could otherwise be spent on paid work or unpaid activities such as caring or food production (subsistence agriculture). In Liberia, CDDs’ financial compensation is dependent on the availability of funds [9, 10], and hence direct and opportunity costs incurred may not be covered, presenting a barrier to their participation in MDA.

This paper presents an analysis of accessibility and adherence to MDA in the post-EVD context from the household, CDD and health system perspective in Liberia. Its findings will help support the Liberian government and partners to move towards equitable attainment of NTD elimination goals and will be of interest to policymakers and implementers facing similar challenges and wishing to develop resilient health systems [8].

The study objective was to document and analyse factors that affect accessibility and adherence to MDA on the demand-side (households) and supply-side (health system) in Liberia. Specific aims were to (i) determine household accessibility and adherence to MDA; (ii) document and quantify the direct and opportunity costs of CDD participation in MDA; and (iii) describe resources used in conducting MDA programs at the community level, and identify demand- and supply-side challenges that adversely affect MDA delivery.

## Methods

### Study components

We addressed the study aims through the following four components: household survey on MDA accessibility and adherence (HHS) (aim 1); CDD costs survey (CDD CS) (aim 2); CDD observational (time and motion) survey during household MDA delivery (CDD TM) (aim 2); key informant surveys with health workers, community health workers and community leaders to explore community-level resources needed for MDA delivery (KIS) and a qualitative synthesis of findings (Aim 3).

### Sampling strategy

For all four components, we purposively selected two counties where all three diseases of interest (LF, onchocerciasis and schistosomiasis) are endemic, where the population resides in predominantly rural areas (reflecting the majority of the Liberian population) and from different geographical regions of Liberia. The selected counties, Bong in Central Liberia and Maryland in South-east Liberia, have similar levels of household wealth across all quintiles, although Bong has higher, and Maryland lower, illiteracy levels compared to the national average [9]. Bong had higher EVD cases than Maryland, with health systems and communities consequently more badly affected by the epidemic. The NTD burden, and geographic and therapeutic program coverage also varies between the two counties.

**Fig. 1:**
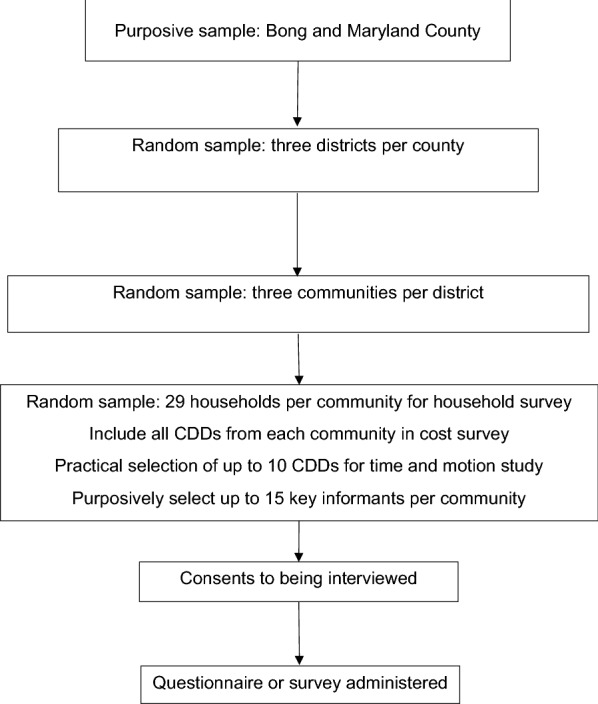
Study sampling frame

Within each county, we used a multi-stage sampling process (Fig. 1) [13]. First, we randomly selected three districts, then we randomly selected three communities from each district, yielding nine communities per county. Once communities had been identified, the sample selection process was different for each study component. For the HHS, we obtained a list of all households from the community head and randomly selected 29 households per community. CDDs are attached to the community with each one covering a 5-km radius for their work. We planned to select all CDDs from the 18 communities for inclusion in the CDD CS and to identify up to 10 CDDs (six Bong, four Maryland) to participate in the CDD TM, based on those actively conducting MDA during the time that the research team was in the community. For component four (KIS), we aimed to select up to 15 key informants per county, including health workers and community leaders. We aimed to include different types of health workers of different genders and experiences within practical constraints such as availability and willingness to participate. The target sample size and the number achieved are shown by study component in Table 1.

**Table 1:** Respondent type, inclusion criteria, target and actual sample size by study component

Study component	Respondent type	Inclusion criteria	Sample size					
			Target			Actual		
			Bong	Maryland	Total	Bong	Maryland	Total
1 Household MDA access and adherence survey (HHS)	Household members	Lived in community during last MDA round^a^	260	260	520	261	263	524
3 CDD cost survey (CDD CS)	CDDs	Assigned to the sampled community	15	14	29	9	15	24
2 CDD time and motion study (CDD TM)	CDDs	Participated in last MDA round	15	14	29	6	4	10
4 Key informant survey (KIS)	Health workers, community health workers and community leaders	Works in/assigned to the sampled community	18	12	30	15	13	28

^a^Last MDA round included Onco and LF (Maryland) and LF (Bong)

### Data collection and analysis

We assigned a team leader and four enumerators to one community at a time to collect data for each study component on Android tablets using Survey CTO, between November 2017 and June 2018.

#### Household survey (HHS)

We conducted interviews with consenting household heads using a questionnaire containing open and closed-ended questions to collect data on knowledge about NTDs and MDA; accessibility and adherence to MDA; MDA drug acceptance; and information sources on MDA. Survey data were analysed to produce indicators of NTD awareness (heard or not heard of NTDs); MDA awareness (as for NTD); whether the respondent took MDA tablets in the last round and if not why; and to identify the most important sources of information on MDA by county and gender.

#### Capturing and analysing CDD costs (CDD CS and CDD TM)

For the CDD CS, we used a questionnaire containing open and closed-ended questions on involvement in MDA activities including CDDs role and experience in training, collection and distribution of drugs, costs incurred, days worked in the MDA, monthly salaries for their main occupation and any allowances received for MDA participation. Direct costs incurred by CDDs during the last MDA round were calculated by summing all reported expenditure on food and transportation costs during MDA training and drug distribution. Direct costs, minus allowances reported, were used to calculate net direct costs [14]. CDD opportunity cost was calculated by multiplying the number of days worked on MDA activities by the value of their time. CDD time was valued using three alternative indicators. Firstly, we used the CDDs' reported wage or salary for their main occupation to impute their daily wage (imputed daily wage [IDW]). We also obtained data on the Liberian minimum wages for unskilled workers and, separately, civil servants [15] which we considered skilled workers. These rates were converted to two daily wage rates, minimum daily wage unskilled (MDWU) and skilled (MDWS). The total cost to CDDs per MDA round was calculated by summing the net direct costs and the estimated opportunity cost [14]. Results are presented as means and medians for the sampled CDDs.

The CDD TM was an observational study where the enumerators followed consenting CDDs during their household rounds using a stopwatch to record the time spent on each separate task in each household. Tasks were coded into a sub-set of the five most reported tasks and then used to calculate the time spent on different tasks, the mean time and CDD opportunity cost per house visited using MDWU and MDWS.

All cost data were in Liberian dollars and converted to US dollars using the 2018 exchange rate (1 LRD = 0.0050 USD) [16]. We cleaned and analysed all quantitative data in Excel [17]

#### Qualitative data collection and analysis (KIS)

The KIS included questions about the organisation and management of the MDA in the communities with a focus on resource use and constraints. Both the KIS and CDD CS contained open-ended questions which enabled respondents to provide free-text responses. In addition, the survey drew on the experiences and comments of the researcher, including critical reflections from their interactions with the CDDs' key informants. We collated and reviewed all free-text data for key themes related to resources needed to implement MDA. We used a framework approach to identify emerging themes from the open-ended questions and critical reflection data [18]. This involved (i) reviewing the data to identify emerging themes, both inductively and deductively, based on emerging findings from the quantitative analysis; (ii) generating a coding framework to apply to the data based on emerging themes; (iii) applying the coding framework and charting the data to identify synergies between themes; and (iv) synthesising findings at a higher level linked to demand- and supply-side factors as presented in the results [18] (Additional file 1: Table S1).

## Results

### Descriptive statistics

For the HHS, we obtained 524 (261 Bong, 263 Maryland) household head interviews (51% male, 49% female) with just over half (53%) coming from the 26–49 age group. Twenty-four CDDs (nine Bong, 15 Maryland) completed the CDD CS, with females making up 25% of the total sample. Ten CDDs (six Bong, four Maryland) were observed for the CDD TM with only one of these being female. Twenty-eight respondents (15 Bong, 13 Maryland) completed the KIS with 39% female (Table 2).

**Table 2:** Descriptive statistics by study component

Study component	Indicator	Category	Bong		Maryland		Total	
			*N*	%	*N*	%	*N*	%
1. Household MDA access and adherence survey (HHS)	Gender	Male	130	50	136	52	266	51
		Female	131	50	127	48	258	49
		Total	261	100	263	100	524	100
	Age	18–25	20	8	11	4	31	6
		26–49	136	52	140	53	276	53
		Over 49	74	28	85	32	159	30
		Not disclosed	31	12	27	10	58	11
		Total	261	100	263	100	524	100
	Disability status^a^	Disability	28	11	26	10	54	10
		No disability	233	89	237	90	470	90
		Total	261	100	263	100	524	100
2. CDD cost survey (CDD CS)	Gender	Male	8	88.9	10	66.7	18	75
		Female	1	11.1	5	33.3	6	25
		Total	9	100	15	100	24	100
	Occupation	Farming	4	44.4	2	13.3	6	25
		Forest ranger	0	0	1	6.7	1	4.1
		Retired nurse	1	11.1	0	0	1	4.1
		Student	0	0	6	40	6	25
		Teacher	2	22.2	2	13.3	4	16.7
		Volunteer	1	11.1	3	20	4	16.7
		Business	1	11.1	1	6.7	2	8.3
		Total	9	100	15	100	24	100
3. CDD time and motion study (CDD TM)	Occupation	Farming	2	33.3	1	30	3	30
		Forest ranger	0	0	1	0	1	10
		Retired nurse	1	16.7	0	20	1	10
		Student	1	16.7	1	0	2	20
		Teacher	0	0	1	0	1	10
		Volunteer	1	16.7	0	50	1	10
		Business	1	16.7	0	0	1	10
		Total	6	100	4	100	10	100
4. Key informant survey (KIS)	Gender	Male	9	60	8	62	17	61
		Female	6	40	5	38	11	39
		Total	15	100	13	100	28	100
	Role	Town chief	5	33	4	31	9	32
		District Surveillance Officer	1	7	0	0	1	4
		Community Health Surveillance Supervisor	4	27	5	38	9	32
		Officer in charge	5	33	1	8	6	21
		Community Development Chairperson	0	0	3	23	3	11
		Total	15	100	13	100	28	100

^a^Disability status was defined using the Washington group of questions on the difficulty in carrying out basic activities (seeing, hearing, walking, self-care, cognition and communication)

### Household accessibility and adherence to MDA (HHS)

More respondents were aware of MDA than NTD in both counties (heard of NTD 13% vs heard of MDA 44%, and 4% vs 25% Bong and Maryland, respectively). Awareness of NTD in Bong was 8% among males and 17% among females, while in Maryland, it was 5% and 2%, respectively. Awareness of MDA in Bong was 43% and 46% (males and females, respectively) and 24% and 26%, respectively, in Maryland. (It is important to note the above results reflect the diverse ways that NTDs and MDA are referred to in the communities and that MDA was a relatively new concept for some communities, e.g., schistosomiasis in Maryland; this diversity may not have been fully reflected in our standardised survey tools.) A lower proportion of males and females reported taking tablets during MDA in Bong than Maryland (Bong 40% male and 40% female, vs Maryland 57% and 55%, respectively). The main reason for males and females reporting not taking the tablet in Bong was not being informed about the MDA activities (male = 22%, female = 24%, both sexes = 23%). In Maryland, for males, it was due to not being informed (9%) or being absent during the MDA activities (female = 20% and both sexes = 14%). The most cited sources of MDA information in both counties (and sexes) were town criers (Table 3).

**Table 3:** Knowledge of and adherence to NTDs and MDA for Bong and Maryland, Liberia

Knowledge/adherence	Responses and sub-totals	Bong						Maryland						Grand total	
		Male		Female		Total		Male		Female		Total			
		*N*	%	*N*	%	*N*	%	*N*	%	*N*	%	*N*	%	*N*	%
NTD awareness	Heard of NTD	11	8	22	17	33	13	7	5	3	2	10	4	43	8
	Not heard of NTD	100	77	101	78	201	77	108	79	120	94	228	87	429	82
	Missing^b^	19	15	6	5	27	10	21	15	4	3	25	10	52	10
	Total	130	100	129	100	261	100	136	100	127	100	263	100	524	100
MDA awareness	Heard of MDA	56	43	59	46	115	44	33	24	33	26	66	25	181	35
	Not heard of MDA	55	42	64	50	119	46	81	60	89	70	170	65	289	55
	Missing^b^	19	15	6	5	27	10	22	16	5	4	27	10	54	10
	Total	130	100	129	100	261	100	136	100	127	100	263	100	524	100
Took tablets during recent MDA round	Yes	52	40	51	40	103	39	77	57	70	55	147	56	250	48
	No	Reason for not taking tablet during recent MDA round													
	Absent	16	12	10	8	26	10	11	8	25	20	36	14	62	12
	Do not know	2	2	2	2	4	2	9	7	8	6	17	6	21	4
	Not informed	28	22	31	24	59	23	12	9	4	3	16	6	75	14
	Pregnant	0	0	7	5	7	3	0	0	5	4	5	2	12	2
	Sick	0	0	1	1	1	0	1	1	1	1	2	1	3	1
	Other	13	10	22	17	35	13	6	4	12	9	18	7	53	10
	Missing^b^	19	15	5	4	26	10	20	15	2	2	22	8	48	9
	Total	130	100	129	100	261	100	136	100	127	100	263	100	524	100
Source of information on MDA^a^	Town crier	81	59	81	60	162	59	60	41	72	59	132	49	294	54
	Radio	32	23	33	24	65	24	35	24	18	15	53	20	118	22
	Hospital	25	18	21	16	46	17	52	35	32	26	84	31	130	24
	Total	138	100	135	100	273	100	147	100	122	100	269	100	542	100

^a^Some respondents cited more than one option, which is why totals are different from other indicators

^b^Missing or no data

### Estimated costs incurred by CDDs throughout the MDA round

The mean reported direct costs incurred by CDDs during the MDA round was $4.17 (median $2.00, range $0.00 to 15.00). Reported mean allowance received per CDD/round was $15.27 ($16.50, $0.00 to $30.00), resulting in a mean net income of $11.10 per CDD/round ($8.13, −$4.00 to $30.00). Mean reported CDD monthly salary was $29.27, yielding an imputed daily wage rate of $1.13 ($0.04, $0 to $12.12). Multiplying the mean reported number of days spent on MDA activity per round per CDD (mean 16.04, median 14, range 2 to 33) by the imputed daily wage rate, minimum daily wage for unskilled and, separately, skilled workers, yields estimated mean indirect costs incurred per CDD per MDA round of $23.00 ($0.27, $0.00 to $169.62), $12.90 ($11.26, $1.61 to $26.53) or $23.15 ($20.20, $2.89 to $47.62). Subtracting the indirect costs from the net direct income (or expenditure if negative) shows that mean cost to CDDs of participating in the MDA round was −$11.90 ($5.04, −$169.62 to $30.00), −$1.79 ($3.12, −$24.53 to $22.37) or −$12.05 (−$6.15, −$45.62 to $17.9) using the IDW, MDWU and MDWS time valuation methods (negative sign implies cost, positive is income gain), respectively (Table 4).

**Table 4:** Estimated costs incurred per CDD during the MDA in Bong and Maryland, Liberia (US$)

	Reported direct costs incurred	Reported allowance received	Net direct income	CDDs reported monthly salary (MS)	Imputed daily wage (IDW)	Reported days spent on MDA activity	Estimated opportunity cost (IDW)	Estimated opportunity cost (MDWU)	Estimated opportunity cost (MDWS)	Cost to CDD (IDW)	Cost to CDD (MDWU)	Cost to CDD (MDWS)
Range	0 to 15	0 to 30	−4 to 30	0 to 315	0 to 12.12	2 to 33	0 to 169.62	1.61 to 26.53	2.89 to 47.62	−169.62 to 30	−24.53 to 22.37	−45.62 to 17.9
Total	100.00	366.50	266.50	702.38	27.01	385.00	552.09	309.54	555.58	−285.59	−43.04	−289.08
SD	5.17	12.38	10.29	66.74	2.57	10.11	44.72	8.13	14.60	49.76	14.20	19.28
Mean	4.17	15.27	11.10	29.27	1.13	16.04	23.00	12.90	23.15	−11.90	−1.79	−12.05
95% CI	3.44 to 4.89	13.54 to 17.00	9.66 to 12.54	19.93 to 38.60	0.77 to 1.48	14.63 to 17.46	16.75 to 29.26	11.76 to 14.04	21.11 to 25.19	−18.86 to −4.94	−3.78 to 0.19	−14.74 to −9.35
Median	2.00	16.50	8.13	1.00	0.04	14.00	0.27	11.26	20.20	5.04	3.12	−6.15
IQR	0.00 to 10.00	4.63 to 30.00	2.75 to 20.00	0.00 to 28.75	0.00 to 1.11	7.00 to 23.25	0.00 to 18,75	5.63 to 18.69	10.10 to 33.55	−13.75 to 17.83	−13.22 to 8.74	−24.73 to −0.12

IDW Imputed daily wage rage is calculated by dividing reported monthly salary by 26 working days per month

MDWU Daily minimum wage rate for unskilled workers is US $0.804. Calculated based on a minimum hourly wage for unskilled workers of 15 Liberian dollars (from https://wageindicator.org/salary/minimum-wage/liberia (accessed 28/07/21)) multiplied by 8 (assuming 8 working hours per day) and converted to US dollars using the June 2018 exchange rate of 0.0067 (from https://live.laborstats.alaska.gov/cpi/calc.cfm (accessed 29/07/21))

MDWS Daily minimum wage rate for skilled workers is US $1.4431. Calculated based on a minimum monthly wage rate for civil servants of 5600 Liberian dollars from https://wageindicator.org/salary/minimum-wage/liberia (accessed 28/07/21) divided by 26 days (per working month) and converted to US dollars as above

Estimated opportunity cost = IDW, MDWU and MDWS multiplied by MDA days

### Household and opportunity costs incurred by all observed CDDs per household visited during MDA (CDD TM)

We observed 10 CDDs for a total of 4645 min in which they undertook 271 household visits to deliver MDA. CDDs conducted a range of tasks with ‘Measuring and drug administration’ taking the most time (mean 8.24 min) followed by ‘record-keeping and registration’ (mean 5.14 min). The mean time per household visited was 17.14 min. (Table 5). Using the MDWU and MDWCS to value this time yields an opportunity cost to the CDD of $0.03–0.05 per house visited.

**Table 5:** Time and opportunity cost by CDDs per MDA household, in Bong and Maryland, Liberia

CDD identifier	Task (time in minutes)						Total time (all tasks)	House visits observed (*n*)
	Measuring and drug administration	Walking	Greetings	Health/awareness talks	Record- keeping and registration	Other		
A	209	0	105	49	217	19	599	25
B	422	2	36	117	133	22	732	25
C	241	48	0	61	176	0	526	29
D	271	9	3	1	147	0	431	30
E	354	18	37	102	101	7	619	27
F	114	28	0	40	114	7	303	25
G	271	69	7	40	272	7	666	52
H	156	25	2	79	115	11	388	29
I	182	41	5	17	114	0	359	28
J	12	0	1	5	4	0	22	1
Total (all CDDs)	2232	240	196	511	1393	73	4645	271
Total time per task (mean)	223.2	24	19.6	51.1	139.3	7.3	464.5	27.1
Time per household by task (mean)	8.24	0.89	0.72	1.89	5.14	0.27	17.14	na
Opportunity cost MDWU (mean) ($)	0.01	0.001	0.001	0.003	0.008	0.0004	0.03	na
Opportunity cost MDWCS (mean) ($)	0.02	0.002	0.002	0.005	0.015	0.0008	0.05	na

NB: stopwatch data were recorded in full minutes; na: not applicable. A–J represent anonymised individual CDDs

### Qualitative findings on community-level resource use and constraints for MDA delivery (KIS)

Key informants in both counties reported that drugs for MDA were supplied by the government and non-governmental organisations (NGOs) and confirmed that the communities bore no costs for drug purchase and transportation. Bong reported the use of wooden and cemented buildings for community-level MDA meetings, while Maryland reported no buildings used for the running of the MDA program. Bong reported the use of three NGO-rented motorcycles during MDA, while Maryland reported using two government-rented motorcycles. Other equipment used in both counties included generators, flip charts, audio and video equipment, megaphones and measuring sticks.

Respondents cited several demand-side challenges to effective MDA such as the important role of community leaders in supporting MDA and identified challenges around expectations for communities to provide ‘compensation’ to CDDs, concerns over side effects, and trust issues. On the supply side, challenges to effective MDA delivery included shortages and delays in medicine availability; frustration among CDDs particularly concerning costs and poor compensation; and reporting challenges (Additional file 1: Table S1) [19].

## Discussion

This study used mixed (quantitative and qualitative) methods to identify and explore community (demand-side) and health system (supply-side) challenges which could potentially affect MDA coverage.

Our results showed low levels of awareness about MDA and lower levels of awareness of NTDs in both counties. We found that 48% of people reported taking the MDA tablets during the last round, with the most common reasons for not adhering being because they were not around when it was being delivered or were not informed about the delivery. This emphasises the importance of ensuring MDA timing and organisation is cognisant of daily and seasonal activities in specific communities [19–22]. As part of the capacity- and awareness-building target of the NTD roadmap to 2030, awareness-generation activities to educate and inform the endemic communities are deemed essential [10], and positive community leader influence and community trust is vital for effective healthcare delivery [23]. Indeed, we found that community leaders encouraged the community to listen to the message from town criers, which was where most respondents reported getting information from in the household survey. More could be done to enhance the role of community leaders in supporting MDA, which should, in turn, generate trust and community ownership of MDA, with likely benefits for coverage and adherence [24–26].

We found that the Liberian CDDs spent an average of 16.04 workdays in the MDA round, similar to the 13.31 workdays a year on NTD activities including MDA in Uganda [27]. Our quantitative findings indicate that Liberian CDDs incurred direct and opportunity costs for taking part in MDA and that for some CDDs, these costs were not adequately compensated by allowances, resulting in a mean cost to CDDs of $11.90. Placing this into context, the minimum monthly wage for civil servants in Liberia is $37.52 (5600 Liberian dollars). This led to frustration among CDDs, which were reflected in our qualitative findings and have been identified in other settings [11, 26–28].

From our KIS survey, we discovered that communities were expected to compensate CDDs via providing gifts in kind such as a cup of rice or a small fee in exchange for medicines to CDDs. Some health staff described that community members were often unable or unwilling to do this due to their level of income and/or socio-economic status and so avoided the CDD. With the high incidence of extreme poverty in the communities receiving MDA and possible deterrence [29–31], this leads us to question the viability of such an approach on equity and efficiency grounds and a potential risk to the achievement of NTD targets on equitable access to healthcare [10]

Our observations of CDDs during their MDA activities identified that two tasks accounted for a large proportion of their time, namely measuring and drug administration, and record-keeping and registration. However, qualitative findings indicated that transportation for CDDs was also a challenge, and they found it difficult to move within communities to distribute drugs. Motorcycles were limited and insufficient to reach out and serve communities, making the workload greater for CDDs as they walked longer distances, taking up more time to distribute drugs and incurring higher direct and opportunity costs by the end of the MDA. Support to compensate CDDs for the costs incurred during their role in MDA delivery and for more efficient transport would likely bring benefits in terms of greater satisfaction and retention, supporting the NTD road map for 2021–2030 [10].

Creating awareness and educating households on MDA and its benefits and side effects of drugs was relatively time-consuming for CDDs at each household. Hence, low awareness increased the workload and consequently the opportunity cost incurred by the CDDs in our study and in others [21, 26]. CDDs could be better supported during MDA with access to materials to support conversations about the reasons for the MDA and drug safety information to reassure households [24, 32, 33].

Having an adequate supply of quality-assured medicines and an efficient supply chain at the community level is critical for the effective allocation and distribution of medicines. However, as in other studies, we found that drug shortages made it difficult for CDDs to effectively distribute drugs to the target population [34]. Similarly, delayed distribution due to drug shortages confused both CDDs and the communities, hampering adherence when the MDA happened [24]. Furthermore, even though most CDDs attended at least one training session, many had limited training on how to complete reporting forms, and consequently, they submitted incomplete records which in turn compromised programmatic-level medicine estimates [35]. These findings highlight the critical importance of NTD monitoring and evaluation mechanisms and suggest that investment in improved CDD training may yield programmatic benefits [10].

Recall bias is inherent in any study relying on household surveys, and this study is no exception. The recruitment of female CDDs was challenging due to fewer females acting as CDDs in the study communities because of pre-existing gender norms, competing domestic priorities, and reduced literacy levels compared to their male counterparts [29, 30]. We also faced capacity challenges that affected the quality of some of the data collected. Analysis of the household survey results on NTD and MDA awareness revealed many missing or no responses. On investigation, it was found that this was due to confusion and lack of understanding of the diverse range of definitions and local terminologies used to refer to NTDs and MDA in the different communities. Unfortunately, due to internet connectivity problems and the community process of data collection, this was not picked up until it was too late to alter the survey tool. This may have led to an underestimate of NTD and MDA awareness in our study.

MDA activities were measured in this study by following the CDDs and using a stopwatch to record time spent on each activity. Unfortunately, time spent by CDDs in houses was only recorded in minutes (not minutes and seconds) which may have affected results. In Uganda, a similar study used pictures and drawings to describe the entire day of the CDD during NTD activities, bringing in more context to the opportunity costs borne by these CDDs [27]. This approach might have worked well in the Liberian context; however, it would not have yielded a quantitative estimate of costs per house, highlighting the benefits of complementary quantitative and qualitative research in understanding health systems.

The data collection limitations of this study highlight the need for continued investment in health research capacity strengthening in low- and middle-income countries. Nevertheless, this work represents a first effort at conducting a mixed-methods health economic study of NTDs in Liberia and thus a major step forward in health systems research for the country.

## Conclusion

Community drug distributors are the interface between the supply- and demand-sides of the health system during MDA and appear to face challenges from both sides. Improved remuneration to meet opportunity cost of MDA participation would likely help to address this and further strengthening the community-level health systems.

## Supplementary Information

**Additional file 1: Table S1.** Supply- and demand-side challenges identified by CDDs, key informants in free-text survey responses and via researcher observations, Bong and Maryland, Liberia.

## Data Availability

The dataset supporting the conclusions of this article is available on request from the authors.

## Abbreviations

NTD: Neglected tropical diseases
MDA: Mass drug administration
CDD: Community drug distributors
LF: Lymphatic filariasis
IVM: Ivermectin
ABD: Albendazole
HHS: Household survey
CDD CS: Community drug distributor cost survey
CDD TM: Community drug distributor time and motion study
KIS: Key informant survey
