# Supplementary material for: Factors influencing mass drug administration adherence and community drug distributor opportunity costs in Liberia: a mixed-methods approach
Source: Parasit Vectors. 2021 Oct 28;14:557. doi: 10.1186/s13071-021-05058-w (PMC8555123; doi:10.1186/s13071-021-05058-w)
Supplement: Supplementary file 1 — Additional file 1: Table S1. Supply- and demand-side challenges identified by CDDs, key informants in free-text survey responses and via researcher observations, Bong and Maryland, Liberia. [file 13071_2021_5058_MOESM1_ESM.docx]

## **Table S1: Supply and demand-side challenges identified by CDDs, key informants in free-text survey responses and via researcher observations, Bong and Maryland, Liberia**

| **Theme summary** | **Quotes** |
| --- | --- |
| **Supply-side** |  |
| ***Shortages and delays in medicine availability*** were identified as a key barrier to achieving full community coverage during MDA. Lack of medicines was often observed to be due to inaccurate population counts during medicine requisition or delays in receiving medicines within the community from higher levels of the health system. | *This community was not served entirely because of the lack of drug. (Researcher Observation Notes, Bong, CDD CS)* |
| **High Opportunity Costs** for CDDs was causing ‘*frustration’* amongst them and leaving them de-motivated. Some community members, health staff and CDDs, felt that the lack of incentives and reliance on communities to compensate CDDs was not working and as a result CDDs took MDA campaigns to be a ‘joke’. This was compounded by the fact that other programmes provided community health cadres with better incentives. CDDs also described that they felt unsatisfied at the lack of adequate compensation they received to attend training activities, which often ended up costing them money. | *Respondent was angry that they are working hard and are not getting anything in return. (Researcher Observation Notes, Bong, CDD CS)*  *The CDDs work on other interventions that provide better incentives; so, they take the MDA for a joke. (Officer in Charge, KIS Bong)*  *I also want the government to help us with transportation and feeding to enable us to get far away communities carry out such activities. (CDD, CDD CS Maryland)*  *I have been a CDD for 9 years and it was only this year I received money (US$15.00) for the training. (CDD, CDD CS Maryland)*  *The CDDs complained that they were not treated fairly, during the training CDDs were not fed and were given US$5.00 far less than what they spent to get to the training hall. The dwellers have refused to provide incentives for the CDDs. They have the notion that the CDDs are paid. (Officer in Charge, KIS Bong)* |
| **Reporting Challenges** were identified as CDDs often had limited training on how to complete reporting forms due to short and condensed training periods. Consequently, they often submitted incomplete records which is compromising for the programme in understanding how to re-adjust medicine estimates and to establish accurate coverage. | *The records from the CDDs are most of the time incomplete because they say it's tedious (Officer in Charge, KIS Bong)* |
| **Limited Awareness** amongst community members in advance of the distribution campaign was described as a problem. Health staff and CDDs described this as being due to budget cuts and condensed distribution timelines meaning that there was not enough resources or time to complete proper sensitisation in advance of medicine distribution. | *The community dwellers complain that they're not informed most of the time of the MDA according to the CDD. The support for CDDs should be increased to improve the MDA (Researcher Observation Notes, CDD CS Maryland)* |
| **Demand-side** |  |
| ***Community leader influence*** was central in supporting communities to understand the reason for medicine distribution and in supporting CDDs to encourage community members to swallow medicines. Community leaders were described as achieving this by supporting with awareness activities, working with CDDs to design innovative distribution approaches, such as carrying medicines to the farm when community members were absent from the community, and in reducing medicine shortages in other locations by supporting CDDs with the monitoring of medicine supply and return of surplus to facilities. | *Once the information about the MDA is brought, I encourage the community to partake and adhere to the instructions. The town crier relays the messages. I monitor the MDA at time and take account of the remaining drug. And see to it that the remaining drug is taken back to the health facilities. (Town Chief 1, KIS Bong)*  *When the medicine come the CDD calls me and we decide how the distribution should go. (Town Chief 2, KIS Bong)* |
| **Community compensation** was described as problematic and meant that some community members refused to engage with the MDA campaign and could leave CDDs feeling de-motivated. Community members are encouraged to provide gifts in kind to CDDs e.g. a cup of rice or to pay a small fee to the CDD in exchange for medicines. However, some health staff described that community members were often unable or unwilling to do this and so avoided the CDD. CDDs also felt compromised by this as they were not only unable to complete their own livelihood activities, they received little in return from the community for taking on the role of a CDD. | *The community dwellers deliberately stay away from taking the drug because they don't want to give the CDDs the compensation of $10 Liberian Dollars [0.1-0.2USD] or a cup of rice (Officer in Charge, KIS Bong).*  *The community that we are working for doesn't appreciate us at all. There are times that we can't carry out our personal work because we've to serve the community. The work is very hard and we don't get enough benefit from it. (CDD,CD CS Maryland)*  *The Chief will encourage the dwellers to compensate the CDDs but it's difficult. (CHSS KIS Bong)* |
| **Community trust** was a challenge when CDDs did not poses an identification card. This could lead to lengthening the time CDDs need to complete drug distribution as they had to spend a long time talking to community members to encourage them to swallow the medicines. In other cases, it led to community members refusing to swallow the medicines and so could contribute to reduced therapeutic coverage. | *People can refuse to take the drug because I don't have identification card. However, I will have to talk to them before they can agree to take the drugs. I am kindly asking the government of Liberia through the county health team to please provide us ID card for the next MDA or any health activity (CDD, CDD CS Maryland)* |
| **Side Effects,** particularly where medicine distribution had been disrupted or had moved to community-based delivery of schistosomiasis treatment, were identified as problematic and could lead to community members refusing to swallow the medicines. Some participants felt that increased awareness amongst community members could also alleviate this challenge. | *The intervention is good for the community but there is a need for more awareness; because people are still insisting on not taking the drugs on grounds that the drugs is not safe, and it can treat them bad (Town Chief, KIS Maryland)* |
